# The biological modifications of milk are linked to mental health of mothers of infants affected by bronchiolitis

**DOI:** 10.1192/j.eurpsy.2024.647

**Published:** 2024-08-27

**Authors:** L. Piccirilli, I. Alberti, A. Pistocchi, V. Bollati, G. P. Milani, M. Buoli

**Affiliations:** ^1^Neuroscience and Mental Health, Fondazione IRCCS Ca’ Granda Ospedale Maggiore Policlinico; ^2^Department of Pathophysiology and Transplantation, University of Milan; ^3^Pediatric Unit, Fondazione IRCCS Ca’ Granda Ospedale Maggiore Policlinico; ^4^Department of Medical Biotechnology and Translational Medicine; ^5^Department of Clinical Sciences and Community Health, University of Milan; ^6^Department of Clinical Sciences and Community Health, Department of Clinical Sciences and Community Health; ^7^Department of Neurosciences and Mental Health, Fondazione IRCCS Ca’ Granda Ospedale Maggiore Policlinico, Milan, Italy

## Abstract

**Introduction:**

Breast milk is a dynamic type of nourishment that changes based on the needs of the child. An increasing amount of data suggests that mental health may be an important factor in such modulation. In addition, breast milk contains extracellular vesicles (EVs), which are currently considered an important dynamic system of communication between cells, even of different individuals.

**Objectives:**

Purpose of this article is to investigate whether changes in breast milk in terms of EVs concentrations are related to maternal mental health.

**Methods:**

This is a case-control study for which we enrolled mothers of infants with bronchiolitis (N=33) and mothers of healthy infants (N=13). Breast milk samples were taken and EVs concentrations were quantified. Maternal mental health was assessed by administration of five different psychometric scales: Edinburgh Postnatal Depression Scale (EPDS), State Trait Anxiety Inventory (STAI-S, STAI-T), Barkin Index of Maternal Functioning (BIMF), The Connor-Davidson Resilience Scale 10 items (CD-RISC). Subsequently, scale scores were related to evs concentrations by negative binomial regressions adjusted for case-control.

**Results:**

As maternal resilience increases, the EVs of neutrophilic origin (p=0.0447) and those of endothelial origin (p=0.0078) decrease¹. In contrast, an increased EPDS score is associated with higher levels of B-lymphocyte EVs (p=0.0376). Scores on the STAI-S scale impact many more populations of EVs²: we observed an increased Incidence Rate Ratio (IRR) of neutrophil-derived EVs (p<0.0001), T-lymphocyte- derived EVs (p=0.0214), NK-cell-derived EVs (p=0.0202), T-reg CD4+ CD25+ (p=0.0141) and endothelial marked EVs (p=0.0180). An increase in STAI-T scale scores also was associated with a significant increase in CD177+ neutrophil-derived EVs (p=0.0028) and endothelial-derived EVs (p=0.0111)³.

**Image:**

**Figure:**
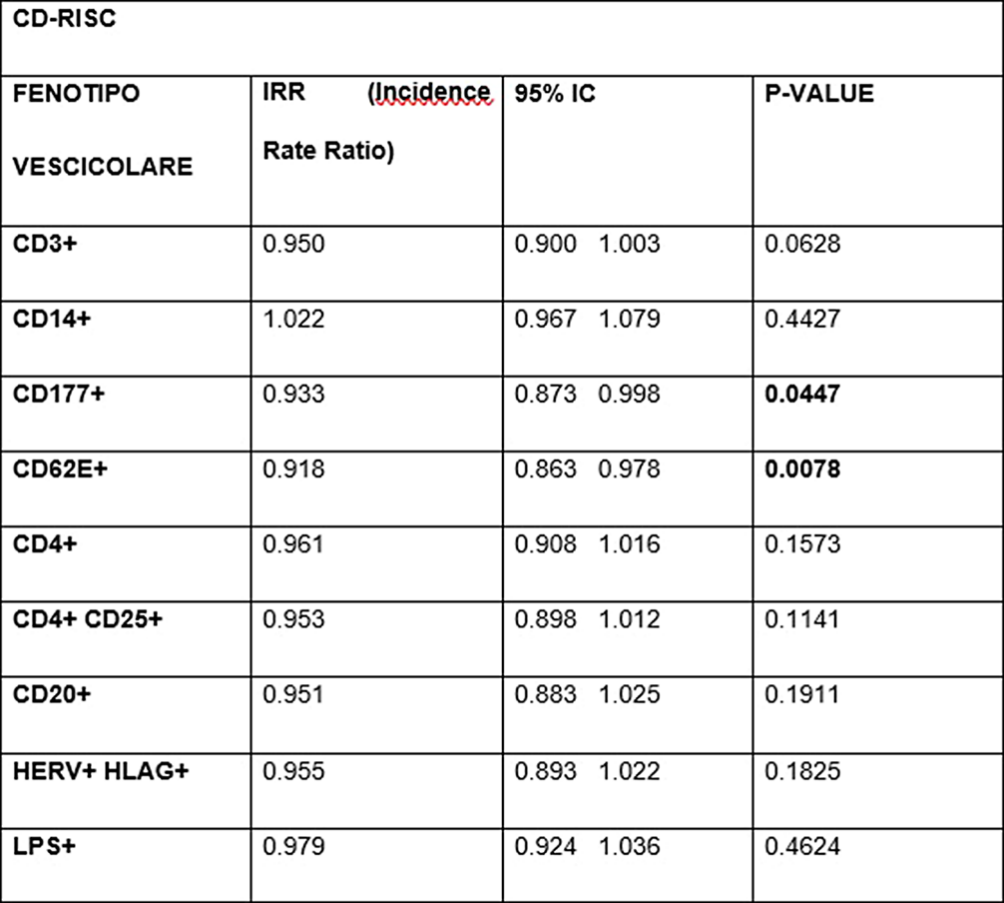

**Image 2:**

**Figure:**
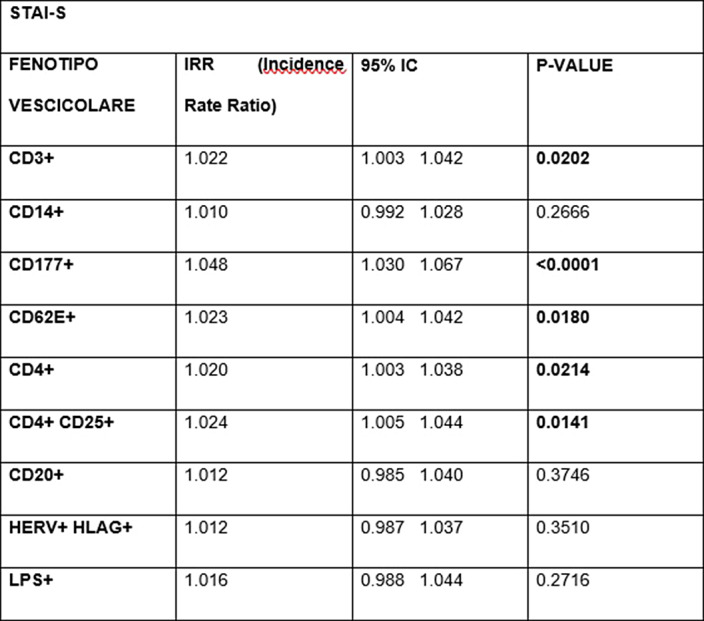

**Image 3:**

**Figure:**
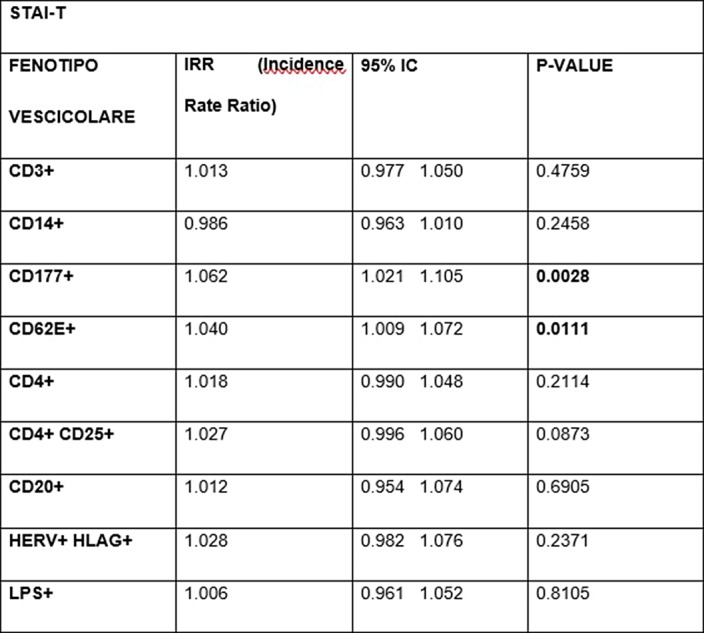

**Conclusions:**

EVs concentrations in breast milk are associated with maternal mental health. Specifically, stress and related severity of anxiety is able to increase the concentrations of EVs derived from inflammatory cells, which suggests an increase in their number and activity. Further research is needed to confirm these preliminary findings.

**Disclosure of Interest:**

None Declared

